# Dynamic weight loss trajectories and machine learning-based prediction of malnutrition in colorectal cancer patients after curative surgery

**DOI:** 10.3389/fnut.2026.1982995

**Published:** 2026-10-21

**Authors:** Xingfa Chen, Jinliang Jian, Zhenmeng Lin, Mingming Xie, Yangming Li

**Affiliations:** 1 NHC key Laboratory of Cancer Metabolism, Department of Radiology, Clinical Oncology School of Fujian Medical University, Fujian Cancer Hospital, Fuzhou, China; 2 NHC key Laboratory of Cancer Metabolism, Department of Colorectal Surgery, Clinical Oncology School of Fujian Medical University, Fujian Cancer Hospital, Fuzhou, China

**Keywords:** colorectal cancer, latent class growth model, machine learning, malnutrition, weight loss trajectory

## Abstract

**Objective:**

This study aimed to identify distinct postoperative weight loss trajectories and develop a machine learning-based model for predicting malnutrition risk at 6 months after curative resection for colorectal cancer (CRC).

**Methods:**

This prospective observational study enrolled 205 CRC patients who underwent curative resection. Weight loss percentages were measured at four postoperative time points relative to preoperative baseline: T1 (postoperative day 7 or hospital discharge), T2 (1 month), T3 (3 months), and T4 (6 months). Latent class growth modeling (LCGM) was employed to identify distinct weight change trajectory patterns. Malnutrition was assessed using the Global Leadership Initiative on Malnutrition (GLIM) criteria at 6 months post-surgery. Core predictors for 6-month malnutrition were screened by taking the intersection of Lasso regression and the Boruta algorithm. Ten machine learning models were constructed and systematically compared, with the optimal model interpreted using SHapley Additive exPlanations (SHAP).

**Results:**

LCGM identified three distinct weight change trajectories: persistent weight loss (28.3%), gradual recovery (38.5%), and rapid recovery (33.2%). Multinomial logistic regression (reference category: persistent weight loss group) showed that age > 65 years and ASA III–IV were associated with lower odds of gradual recovery, whereas colon cancer (right or left) was associated with higher odds. For rapid recovery, female, age > 65 years, ASA III–IV, and adjuvant chemotherapy were associated with lower odds, whereas colon cancer (right or left) was associated with higher odds. The overall incidence of GLIM-defined malnutrition at 6 months was 33.2%. Among the ten machine learning models, XGBoost achieved the best comprehensive performance, with an AUC of 0.836 (95% CI: 0.779–0.894). Calibration curves demonstrated good agreement between predicted and observed probabilities, and decision curve analysis confirmed net clinical benefit across a 9–91% threshold range. SHAP analysis revealed that lower preoperative BMI, receipt of adjuvant chemotherapy, older age, and rectal tumor location were associated with increased malnutrition risk.

**Conclusion:**

This study revealed three distinct postoperative weight loss trajectories in CRC patients. The developed XGBoost model, incorporating four readily available clinical variables, demonstrated favorable predictive performance and clinical utility for early identification of patients at high risk of long-term malnutrition, pending further external validation.

## Introduction

Colorectal cancer (CRC) remains one of the most common malignancies worldwide, ranking third in global incidence and second in cancer-related mortality (1, 2). Over the past decades, advances in early detection, surgical techniques, and effective neoadjuvant/adjuvant therapeutic regimens have substantially improved oncological outcomes, with 5-year survival rates exceeding 70% in patients with non-metastatic disease (3, 4). Consequently, the focus of CRC survivorship care has progressively shifted from disease control alone toward comprehensive functional rehabilitation and long-term quality-of-life preservation (5).

Malnutrition represents a prevalent and serious complication in CRC patients, serving not merely as a nutritional derangement but also as a robust independent predictor of impaired immune function, delayed wound healing, reduced tolerance to adjuvant therapy, diminished physical performance, and ultimately, inferior survival outcomes (6–10).

Nevertheless, the vast majority of existing studies have focused almost exclusively on preoperative nutritional status or short-term perioperative changes, whereas the dynamic process of weight recovery during the extended post-discharge period (spanning several months after surgery) remains markedly under-investigated. Cross-sectional assessments at isolated time points are inherently inadequate to capture the heterogeneous recovery trajectories that patients may follow: some individuals regain their preoperative weight rapidly, while others experience persistent weight loss and fail to recover even by six months postoperatively; yet the drivers of these divergent patterns remain poorly understood. Moreover, although several predictive models for postoperative nutritional deterioration have been developed in other malignancies—notably, machine learning-based models for malnutrition risk prediction have been successfully established in gastric and lung cancer populations and have demonstrated encouraging performance—a robust, dedicated, and externally generalizable prediction tool specifically tailored to CRC patients remains conspicuously absent (11–14). This gap is particularly noteworthy because CRC patients present distinct pathophysiological features compared to these cancer types, including differences in anatomical location, unique functional sequelae of surgical resection (e.g., low anterior resection syndrome [LARS]), and distinct adjuvant chemotherapy regimens—all of which may modulate malnutrition risk through mechanisms that models derived from other tumor types cannot fully capture.

To address these gaps, this prospective study applied latent class growth modeling to delineate heterogeneous postoperative weight recovery trajectories and employed machine learning to construct a CRC-specific malnutrition prediction model, aiming to facilitate early risk stratification and personalized nutritional management for high-risk survivors.

## Methods

### Patients

This prospective observational study was conducted at Fujian Cancer Hospital between September 2024 and April 2025. Consecutive patients who underwent curative resection for primary colorectal adenocarcinoma were enrolled. Inclusion criteria were: (1) histopathologically confirmed primary colorectal adenocarcinoma; (2) underwent curative-intent surgery; (3) aged ≥18 years; and (4) had complete clinical data with reliable follow-up, including at least three postoperative weight measurements and an abdominal CT scan at 6 months after surgery. Exclusion criteria were: (1) emergency surgery due to intestinal obstruction, perforation, or other acute conditions; (2) concurrent other malignancies; (3) pre-existing severe hepatic, renal, or cardiac insufficiency; and (4) radiologically confirmed liver metastasis (including oligometastasis), regardless of whether curative resection was performed, to avoid confounding by metastatic disease-related cachexia. Based on these criteria, a total of 205 patients were ultimately included. The patient selection process is detailed in Figure 1.

**Figure 1:**
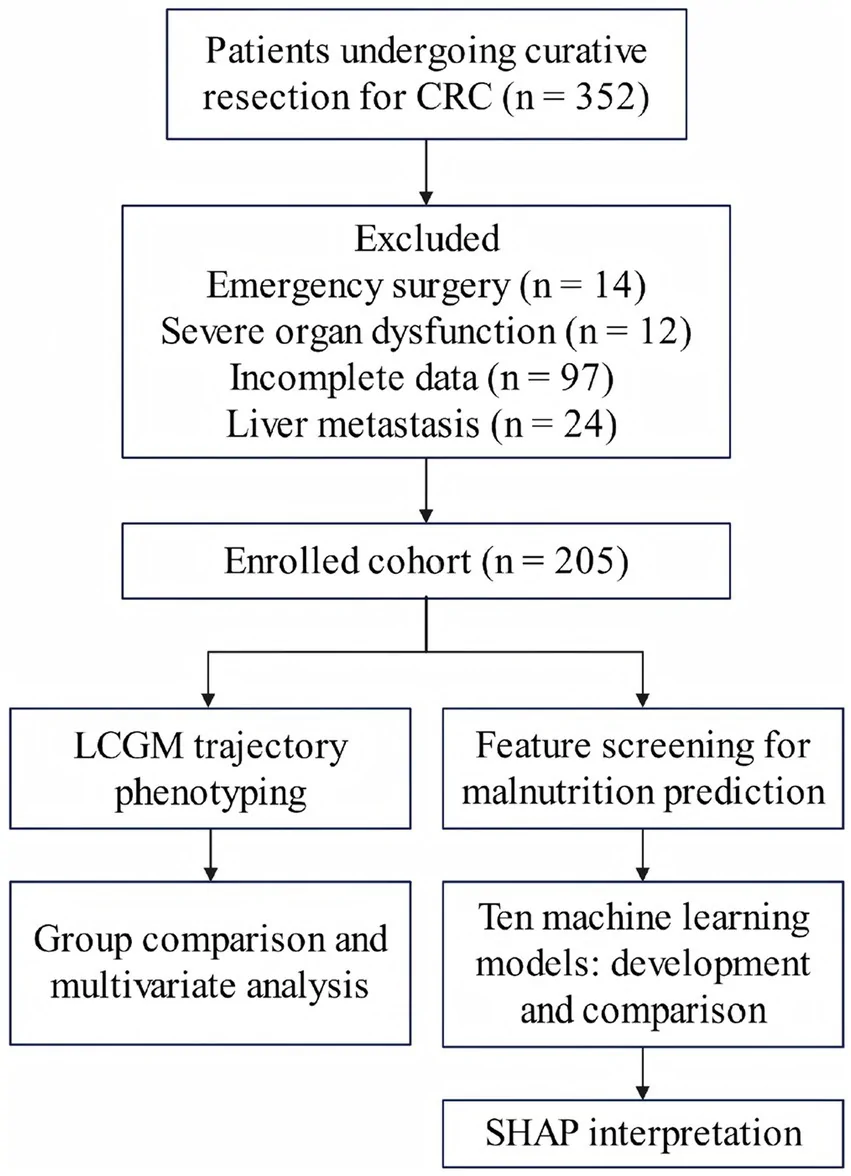
Study flow diagram. CRC, colorectal cancer; LCGM, latent class growth modeling; SHAP, SHapley Additive exPlanations.

### Outcome measures

Body weight was measured at five distinct time points: preoperatively (baseline), and postoperatively at T1, T2, T3, and T4. The postoperative time points were defined as follows: T1, postoperative day 7 (POD7) or the last assessment before discharge if POD7 data were unavailable; T2, 1 month post-surgery; T3, 3 months post-surgery; and T4, 6 months post-surgery. The percentage of weight loss at each postoperative time point was calculated relative to the preoperative baseline using the formula: (preoperative weight – postoperative weight) / preoperative weight × 100%. Postoperative weight data were collected during outpatient follow-up visits or via structured telephone interviews.

Malnutrition at 6 months post-surgery was diagnosed using the two-step approach recommended by the Global Leadership Initiative on Malnutrition (GLIM) consensus. In the first step, nutritional risk screening was performed using the Nutritional Risk Screening 2002 (NRS-2002) tool, with a score ≥3 indicating the presence of nutritional risk. In the second step, the diagnosis of malnutrition required fulfillment of at least one phenotypic criterion and one etiologic criterion. Since all patients in this cohort had colorectal cancer, the etiologic criterion (namely, disease burden/inflammation) was considered inherently satisfied under the GLIM framework. Therefore, patients with an NRS-2002 score ≥3 were diagnosed as malnourished if they met any of the following three phenotypic criteria: (1) involuntary weight loss >5% within the past 6 months (or >10% beyond 6 months); (2) low body mass index (BMI), defined as <18.5 kg/m^2^ for patients aged <70 years or <20 kg/m^2^ for those aged ≥70 years (15); or (3) reduced muscle mass, assessed by computed tomography (CT)-derived skeletal muscle index at the third lumbar vertebra (L3-SMI) (Supplementary Figure 1), with sex-specific cut-offs of <40.8 cm^2^/m^2^ for males and <34.9 cm^2^/m^2^ for females (8, 16–18).

The prediction index date was defined as the time when postoperative pathological results became available (typically 5–7 days after surgery, at or before hospital discharge). All four predictors were assessed at or before this time point: age and preoperative BMI were obtained preoperatively; tumor location was determined by preoperative colonoscopy and CT; and planned adjuvant chemotherapy was determined based on postoperative pathological staging within 5–7 days after surgery. Therefore, all predictors were available at the intended prediction time.

### Statistical analysis

All statistical analyses were performed using SPSS version 27.0, R software version 4.5.2, and Mplus version 8.3. Latent class growth modeling (LCGM) was used to identify distinct weight loss trajectories. Model selection was based on the Akaike information criterion (AIC), Bayesian information criterion (BIC), sample size-adjusted BIC (aBIC), entropy, and the Lo–Mendell–Rubin adjusted likelihood ratio test (LMRT) and bootstrap likelihood ratio test (BLRT); priority was given to parsimony and clinical interpretability among models with comparable or superior statistical indices. Entropy >0.80 indicated good classification accuracy. For the LCGM, missing longitudinal weight data were handled using full information maximum likelihood (FIML) under the missing-at-random (MAR) assumption. To compare weight loss percentages across the four postoperative time points (T1–T4), a linear mixed model (LMM) was employed. The model included time as a fixed effect (modeled as a categorical variable with four levels: T1, T2, T3, and T4) and a random intercept for each patient to account for within-subject correlation. An unstructured covariance matrix was used, and parameters were estimated using restricted maximum likelihood (REML). The LMM used all available observations under the MAR assumption. *Post hoc* pairwise comparisons across time points were performed with Bonferroni correction.

For the latent class growth modeling, multinomial logistic regression, and machine learning analyses, age was dichotomized as ≤65 vs. > 65 years, a widely accepted threshold defining the older adult population in clinical research and geriatric oncology (19, 20). This cutoff is distinct from the age-specific thresholds (<70 vs. ≥70 years) used exclusively for the GLIM low-BMI phenotypic criterion. Between-group comparisons across trajectory classes were performed using one-way analysis of variance (ANOVA) or Kruskal-Wallis tests for continuous variables, and chi-square or Fisher’s exact tests for categorical variables. For variables with a significant overall test (*p* < 0.05), post-hoc pairwise comparisons were further conducted. For continuous variables, Tukey’s test was used when equal variances were assumed; otherwise, the Games-Howell test was applied. For categorical variables, pairwise chi-square tests were performed with Bonferroni correction for multiple comparisons. Because the outcome (weight change trajectory) had three categories, multinomial logistic regression was used, with the persistent weight loss group as the reference category. Variables with *p* < 0.05 in univariate comparisons were entered into the multivariable model to maintain parsimony and avoid overfitting given the modest sample size. Missing data for the other clinical variables were handled using multiple imputation with chained equations (MICE), also under the MAR assumption. The proportion of missing values for each analyzed variable is reported in Supplementary Table 1. Twenty imputed datasets were generated, and pooled estimates were obtained based on Rubin’s rules.

To identify core predictors of 6-month malnutrition, Lasso regression (with 10-fold cross-validation for λ selection) and the Boruta algorithm were applied sequentially within each training fold of the five-fold cross-validation. Specifically, in each fold, feature selection was performed exclusively on the training set, and the final predictors were defined as the intersection of both methods. The selected features were then used to train the machine learning models, which were evaluated on the corresponding held-out validation set. For the final model, the four predictors selected from the full dataset were used. Multicollinearity was assessed using Spearman correlation and variance inflation factor (VIF < 5).

Ten machine learning models were developed and evaluated using five-fold cross-validation: support vector machine (SVM), gradient boosting machine (GBM), neural network (NN), K-nearest neighbors (KNN), naive Bayes (NB), multilayer perceptron (MLP), extreme gradient boosting (XGBoost), random forest (RF), decision tree (DT), and logistic regression (LR). Model performance was compared across discrimination (area under the curve [AUC], sensitivity, specificity, accuracy, precision, F1 score, and negative predictive value [NPV]), calibration (Brier score and calibration curves), and clinical utility (decision curve analysis [DCA]). The optimal model was interpreted using SHapley Additive exPlanations (SHAP). A two-sided *p* value < 0.05 was considered statistically significant.

## Results

### Dynamic postoperative weight change trajectories in colorectal cancer patients

Among the 205 patients included, the overall weight loss percentages at the four postoperative time points (T1, T2, T3, and T4) were 7.8%, 5.6%, 3.5%, and 2.5%, respectively, indicating a progressive decrease in weight loss percentage with subsequent stabilization over time (Supplementary Figure 2). The linear mixed model revealed a significant overall effect of time on weight loss percentage (*p* < 0.001), and *post hoc* pairwise comparisons with Bonferroni correction showed that all pairwise comparisons between time points were statistically significant (all *p* < 0.001).

To identify distinct patterns of postoperative weight change, we fitted LCGM to the longitudinal weight loss data. Model selection was based on a combination of statistical indices (AIC, BIC, aBIC, entropy, LMRT, and BLRT) and clinical interpretability. The 3-class model was ultimately selected as the optimal solution. Although the 4-class solution showed significant LMRT (*p* = 0.001) and BLRT (*p* = 0.001), it was discarded because it had higher AIC and BIC values (indicating poorer fit), a smaller and less clinically distinctive additional subgroup (15.6% of patients), and no clear clinical interpretability. In contrast, the 3-class model achieved excellent entropy (0.993), balanced class proportions, and straightforward clinical interpretability. We therefore prioritized parsimony, lower AIC/BIC, and clinical meaningfulness over the statistical significance of the LMRT/BLRT in choosing the 3-class solution. Based on the trajectory characteristics, the three classes were designated as: the persistent weight loss group (*n* = 58, 28.3%), the gradual recovery group (*n* = 79, 38.5%), and the rapid recovery group (*n* = 68, 33.2%) (Figure 2).

**Figure 2:**
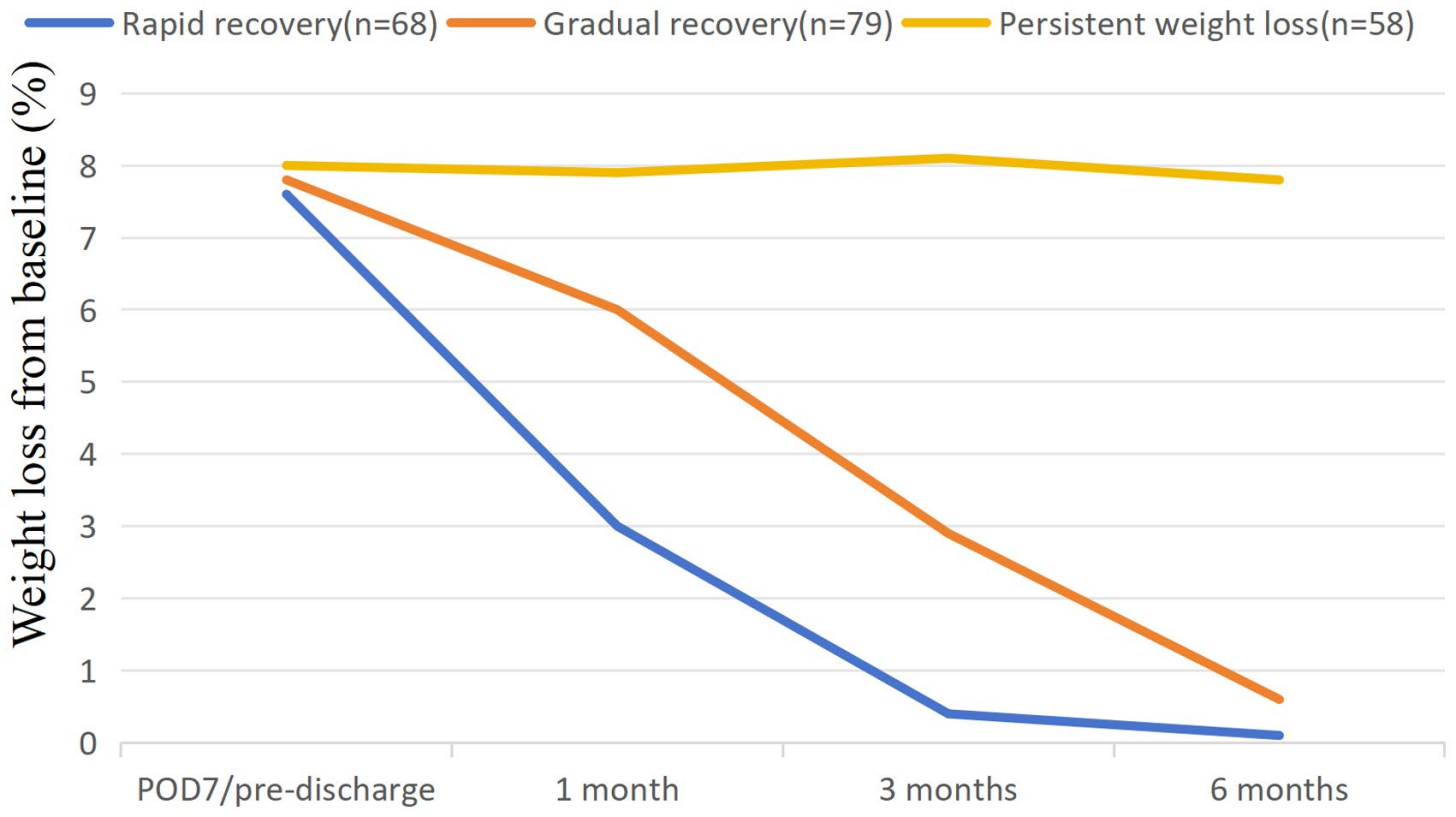
Latent class growth trajectories of weight loss percentage from baseline across four postoperative time points.

In the persistent weight loss group, weight loss remained consistently elevated across all time points, with T1–T4 values of 8.0%, 7.9%, 8.1%, and 7.8%, indicating sustained weight deficit throughout the 6-month postoperative period. In the gradual recovery group, substantial early weight loss was followed by a progressive return toward baseline, with T1–T4 values of 7.8%, 6.0%, 2.9%, and 0.6%. In contrast, the rapid recovery group showed the fastest restitution, achieving near-baseline weight by 3 months post-surgery, with T1–T4 values of 7.6%, 3.0%, 0.4%, and 0.1% (Supplementary Table 2; Table 1).

**Table 1:** Model fit comparison for latent class growth trajectories.

Number of classes	AIC	BIC	aBIC	*p* value		Entropy	Class proportions
				LMRT	BLRT		
1	4261.674	4288.258	4262.911	—	—	—	1
2	3790.701	3833.900	3792.712	0.001	<0.001	0.982	0.288/0.712
3	2874.174	2967.218	2878.504	<0.001	0.001	0.993	0.283/0.385/0.332
4	3023.784	3100.213	3027.341	0.001	0.001	0.998	0.263/0.351/0.156/0.230
5	3317.911	3377.725	3320.695	0.021	0.023	0.999	0.263/0.312/0.254/0.112/0.059

AIC, Akaike information criterion; BIC, Bayesian information criterion; aBIC, sample size-adjusted Bayesian information criterion; LMRT, Lo–Mendell–Rubin adjusted likelihood ratio test; BLRT, bootstrap likelihood ratio test.

### Comparison of clinicopathological characteristics and factors associated with trajectory groups

Univariate analysis was performed to compare the three trajectory groups. Variables with *p* < 0.05 included sex, age, American Society of Anesthesiologists (ASA) grade, operation time, tumor location, postoperative complications, and adjuvant chemotherapy (Table 2). These variables were then entered into a multinomial logistic regression model with the three-category trajectory outcome as the dependent variable, and the persistent weight loss group served as the reference category (Table 3).

**Table 2:** Univariate analysis of clinicopathological characteristics among patients stratified by postoperative weight change trajectories.

Variables	Persistent weight loss (*n* = 58)	Gradual recovery (*n* = 79)	Rapid recovery (*n* = 68)	*p*
Sex ^a, b^				0.011
Male	29 (14.1)	45 (22.0)	51 (24.9)	
Female	29 (14.1)	34 (16.6)	17 (8.3)	
Age (years) ^b^				0.003
≤65	23 (11.2)	50 (24.4)	46 (22.4)	
>65	35 (17.1)	29 (14.1)	22 (10.7)	
Hypertension				0.506
Yes	20 (9.8)	23 (11.2)	17 (8.3)	
No	38 (18.5)	56 (27.3)	51 (24.9)	
Diabetes mellitus				0.122
Yes	12 (5.9)	9 (4.4)	6 (2.9)	
No	46 (22.4)	70 (34.1)	62 (30.2)	
Respiratory disease				0.373
Yes	8 (3.9)	6 (2.9)	5 (2.4)	
No	50 (24.4)	73 (35.6)	63 (30.7)	
Smoking				0.226
Yes	10 (4.9)	9 (4.4)	5 (2.4)	
No	48 (23.4)	70 (34.1)	63 (30.7)	
Alcohol consumption				0.229
Yes	15 (7.3)	13 (6.3)	10 (4.9)	
No	43 (21.0)	66 (32.2)	58 (28.3)	
Previous abdominal surgery				0.664
Yes	7 (3.4)	6 (2.9)	6 (2.9)	
No	51 (24.9)	73 (35.6)	62 (30.2)	
Preoperative BMI (kg/m^2^)	22.0 (20.4, 23.5)	22.7 (20.0, 24.4)	23.1 (20.9, 25.1)	0.079
Neoadjuvant therapy				0.286
Yes	20 (9.8)	18 (8.8)	17 (8.3)	
No	38 (18.5)	61 (29.8)	51 (24.9)	
Preoperative CEA (ng/mL)				0.613
≤5	36 (17.6)	46 (22.4)	45 (22.0)	
>5	22 (10.7)	33 (16.1)	23 (11.2)	
Preoperative CA19-9 (U/mL)				0.225
≤30	46 (22.4)	64 (31.2)	61 (29.8)	
>30	12 (5.9)	15 (7.3)	7 (3.4)	
ASA grade ^b^				0.014
I-II	42 (20.5)	68 (33.2)	62 (30.2)	
III-IV	16 (7.8)	11 (5.4)	6 (2.9)	
Surgical approach				0.129
Laparoscopic	47 (22.9)	61 (29.8)	45 (22.0)	
Robotic	11 (5.4)	18 (8.8)	23 (11.2)	
Operation time (min) ^b^	205.0 ± 47.6	194.5 ± 63.9	181.2 ± 46.3	0.048
Intraoperative blood loss (mL)	61.4 ± 28.4	52.2 ± 24.3	49.6 ± 25.8	0.367
Tumor location ^a, b^				<0.001
Rectum	39 (19.0)	23 (11.2)	19 (9.3)	
Left colon	16 (7.8)	32 (15.6)	32 (15.6)	
Right colon	3 (1.5)	24 (11.7)	17 (8.3)	
Tumor size (cm)	4.6 ± 2.1	4.2 ± 1.7	3.8 ± 2.1	0.086
Pathological stage				0.107
I	14 (6.8)	26 (12.7)	29 (14.1)	
II	18 (8.8)	24 (11.7)	23 (11.2)	
III	26 (12.7)	29 (14.1)	16 (7.8)	
Differentiation				0.601
Well/moderate	50 (24.4)	72 (35.1)	59 (28.8)	
Poor	8 (3.9)	7 (3.4)	9 (4.4)	
Lymphovascular invasion				0.315
Yes	9 (4.4)	11 (5.4)	5 (2.4)	
No	49 (23.9)	68 (33.2)	63 (30.7)	
Stoma				0.162
Yes	12 (5.9)	11 (5.4)	6 (2.9)	
No	46 (22.4)	68 (33.2)	62 (30.2)	
Postoperative complications ^b^				0.005
Yes	15 (7.3)	10 (4.9)	4 (2.0)	
No	43 (21.0)	69 (33.7)	64 (31.2)	
Adjuvant chemotherapy ^b,c^				<0.001
Yes	45 (22.0)	52 (25.4)	28 (13.7)	
No	13 (6.3)	27 (13.2)	40 (19.5)	

Superscript lowercase letters above each variable denote the between-group pairwise comparisons that were statistically significant (*p* < 0.05): a, Persistent weight loss vs Gradual recovery; b, Persistent weight loss vs Rapid recovery; c, Gradual recovery vs Rapid recovery. No letter is shown for a given pair when its difference was not statistically significant. All percentages were calculated relative to the total sample (*n* = 205) rather than within each trajectory subgroup. ASA, American Society of Anesthesiologists; BMI, body mass index; CEA, carcinoembryonic antigen; CA19-9, carbohydrate antigen 19–9.

**Table 3:** Multinomial logistic regression analysis of factors associated with weight trajectory groups.

Trajectory group	Variable	*p*	OR (95% CI)
Gradual recovery group	Sex (ref: Male)	0.559	0.784 (0.347–1.772)
	Age (ref: ≤65)	0.029	0.403 (0.179–0.910)
	ASA grade (ref: I–II)	0.043	0.350 (0.127–0.968)
	Operation time	0.311	0.996 (0.990–1.003)
	Tumor location (ref: rectum)		
	Left colon	0.006	3.432 (1.432–8.229)
	Right colon	0.002	6.368 (2.118–19.479)
	Postoperative complications (ref: No)	0.674	0.803 (0.288–2.239)
	Adjuvant chemotherapy (ref: No)	0.057	0.419 (0.171–1.024)
Rapid recovery group	Sex (ref: Male)	0.039	0.354 (0.131–0.955)
	Age (ref: ≤65)	0.018	0.302 (0.112–0.812)
	ASA grade (ref: I–II)	0.045	0.239 (0.060–0.949)
	Operation time	0.194	0.994 (0.984–1.003)
	Tumor location (ref: rectum)		
	Left colon	0.031	2.966 (1.102–7.979)
	Right colon	0.001	9.443 (2.116–42.166)
	Postoperative complications (ref: No)	0.073	0.249 (0.054–1.140)
	Adjuvant chemotherapy (ref: No)	0.003	0.221 (0.080–0.607)

The persistent weight loss group served as the reference category. Reference categories are indicated in parentheses for each dichotomized variable. Operation time was analyzed as a continuous variable. ASA, American Society of Anesthesiologists.

For the gradual recovery group versus the persistent weight loss group, age > 65 years (OR = 0.403, 95% CI: 0.179–0.910, *p* = 0.029) and ASA grade III-IV (OR = 0.350, 95% CI: 0.127–0.968, *p* = 0.043) were significantly associated with a lower likelihood of gradual weight recovery, whereas left colon cancer (OR = 3.432, 95% CI: 1.432–8.229, *p* = 0.006) and right colon cancer (OR = 6.368, 95% CI: 2.118–19.479, *p* = 0.002) were independent protective factors favoring gradual recovery.

For the rapid recovery group versus the persistent weight loss group, female (OR = 0.354, 95% CI: 0.131–0.955, *p* = 0.039), age > 65 years (OR = 0.302, 95% CI: 0.112–0.812, *p* = 0.018), ASA grade III-IV (OR = 0.239, 95% CI: 0.060–0.949, *p* = 0.045), and adjuvant chemotherapy (OR = 0.221, 95% CI: 0.080–0.607, *p* = 0.003) were negatively associated with rapid weight recovery, whereas left colon cancer (OR = 2.966, 95% CI: 1.102–7.979, *p* = 0.031) and right colon cancer (OR = 9.443, 95% CI: 2.116–42.166, *p* = 0.001) were independent protective factors favoring rapid recovery.

### Screening of predictors for postoperative 6-month malnutrition

Among the 205 patients, 68 (33.2%) were diagnosed with malnutrition at 6 months post-surgery according to the GLIM criteria. To identify core predictors, a combined dimensionality reduction strategy using Lasso regression and the Boruta algorithm was applied to all candidate variables. Lasso regression selected five predictors at λ = 0.0455: age, BMI, tumor location, postoperative complications, and adjuvant chemotherapy—ultimately retained as predictors of 6-month malnutrition. Feature selection was re-performed within each training fold of the cross-validation, and the Lasso—Boruta intersection consistently yielded the same four predictors; the full-dataset analysis presented in Figure 3 refers to the final model.

**Figure 3:**
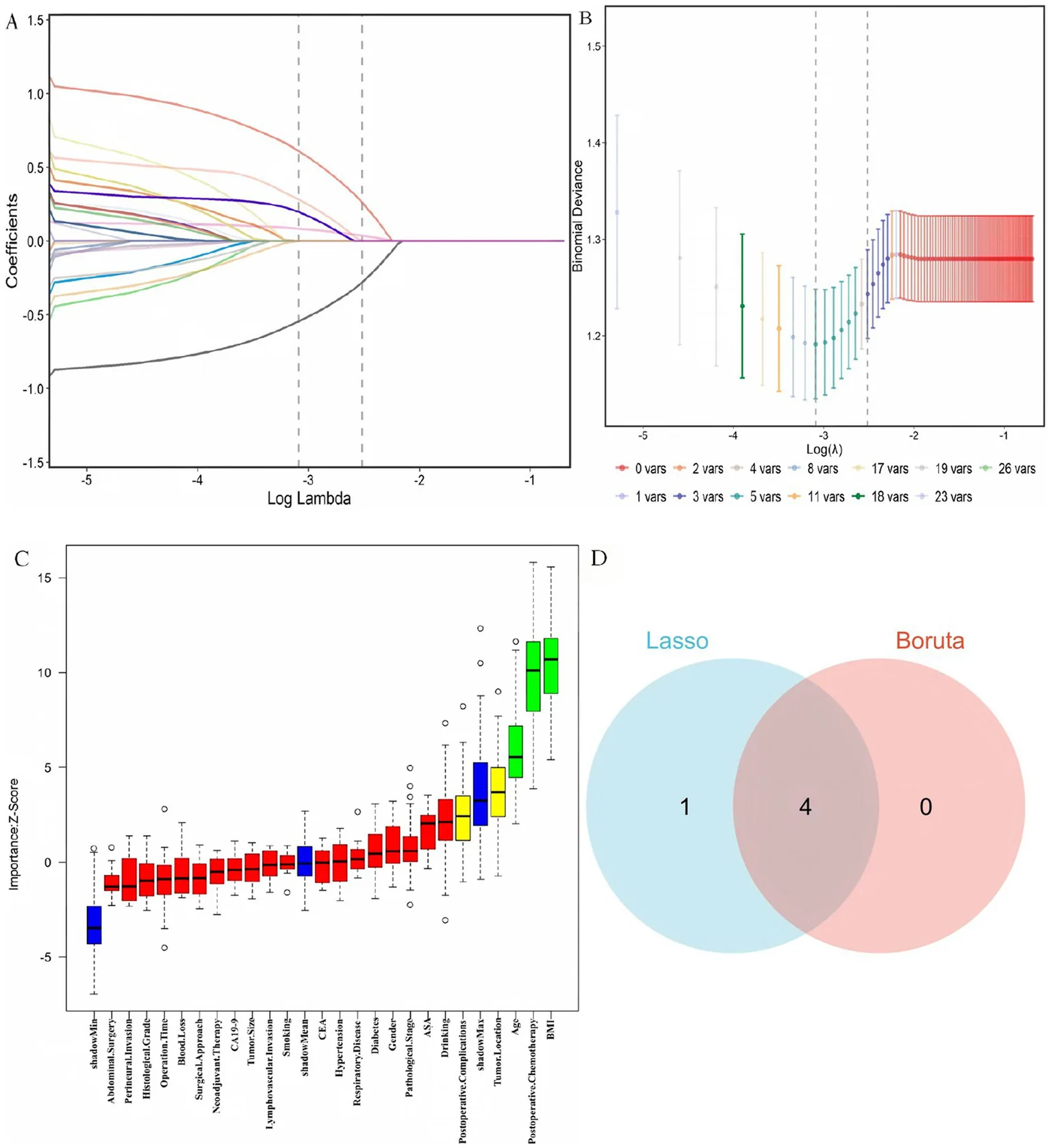
Combined Lasso–Boruta predictor screening. **(A)** Lasso coefficient paths and optimal λ. **(B)** Cross-validation error curve. **(C)** Boruta importance ranking (confirmed/rejected/shadow). **(D)** Venn diagram of selected variables; four overlapping predictors (age, BMI, tumor location, adjuvant chemotherapy) were finalized.

Spearman correlation analysis among the four predictors showed low inter-variable correlations, with all pairwise comparisons being non-significant (all *p* > 0.05), indicating no statistically significant linear relationships (Supplementary Figure 3). Collinearity diagnostics further confirmed the absence of significant multicollinearity, with tolerance values ranging from 0.985 to 0.997 (all > 0.1) and variance inflation factor (VIF) values ranging from 1.003 to 1.015 (all < 5) (Supplementary Table 3).

### Development and evaluation of machine learning prediction models

Based on the four predictors identified by Lasso regression and the Boruta algorithm (age, BMI, tumor location, and adjuvant chemotherapy), ten machine learning models were constructed. The performance metrics of each model are summarized in Table 4.

**Table 4:** Performance comparison of ten machine learning prediction models.

Model	Sensitivity	Specificity	Accuracy	NPV	Precision	F1	AUC	Brier
SVM	0.745	0.799	0.781	0.863	0.648	0.693	0.804 (0.745–0.863)	0.195
GBM	0.691	0.796	0.761	0.838	0.627	0.657	0.776 (0.705–0.846)	0.181
NN	0.642	0.824	0.764	0.823	0.644	0.643	0.772 (0.704–0.841)	0.175
KNN	0.715	0.750	0.738	0.841	0.587	0.645	0.784 (0.715–0.853)	0.191
NB	0.620	0.897	0.805	0.826	0.749	0.679	0.822 (0.764–0.880)	0.105
MLP	0.745	0.765	0.758	0.858	0.611	0.672	0.820 (0.762–0.878)	0.107
XGBoost	0.766	0.779	0.775	0.870	0.632	0.693	0.836 (0.779–0.894)	0.098
RF	0.774	0.721	0.739	0.865	0.579	0.663	0.812 (0.749–0.876)	0.128
DT	0.642	0.809	0.754	0.820	0.625	0.634	0.747 (0.677–0.817)	0.186
LR	0.591	0.853	0.766	0.808	0.666	0.626	0.742 (0.671–0.813)	0.199

Malnutrition was defined as the positive class for all performance metrics. AUC, area under the curve; NPV, negative predictive value; F1, F1 score; Brier, Brier score; SVM, support vector machine; GBM, gradient boosting machine; NN, neural network; KNN, K-nearest neighbors; NB, naive Bayes; MLP, multilayer perceptron; XGBoost, extreme gradient boosting; RF, random forest; DT, decision tree; LR, logistic regression.

Among the ten models, XGBoost demonstrated the best comprehensive performance across multiple evaluation dimensions. It yielded the highest AUC of 0.836 (95% CI: 0.779–0.894), with an accuracy of 0.775, sensitivity of 0.766, specificity of 0.779, and F1 score of 0.693. It also achieved the lowest Brier score of 0.098 among all models, reflecting favorable calibration, and the calibration curve showed good agreement between the predicted probabilities and actual observations. Although NB, MLP, and random forest showed relatively comparable AUC values, XGBoost provided net clinical benefit across a wider threshold probability range of 9–91% according to DCA, indicating more favorable practical clinical utility for postoperative malnutrition risk stratification. Given that the model relies on only four readily available clinical predictors, this combination of discrimination, calibration, and net benefit supports its use for early risk stratification. Considering these factors together, XGBoost was selected as the optimal model for subsequent SHAP interpretability analysis (Figure 4).

**Figure 4:**
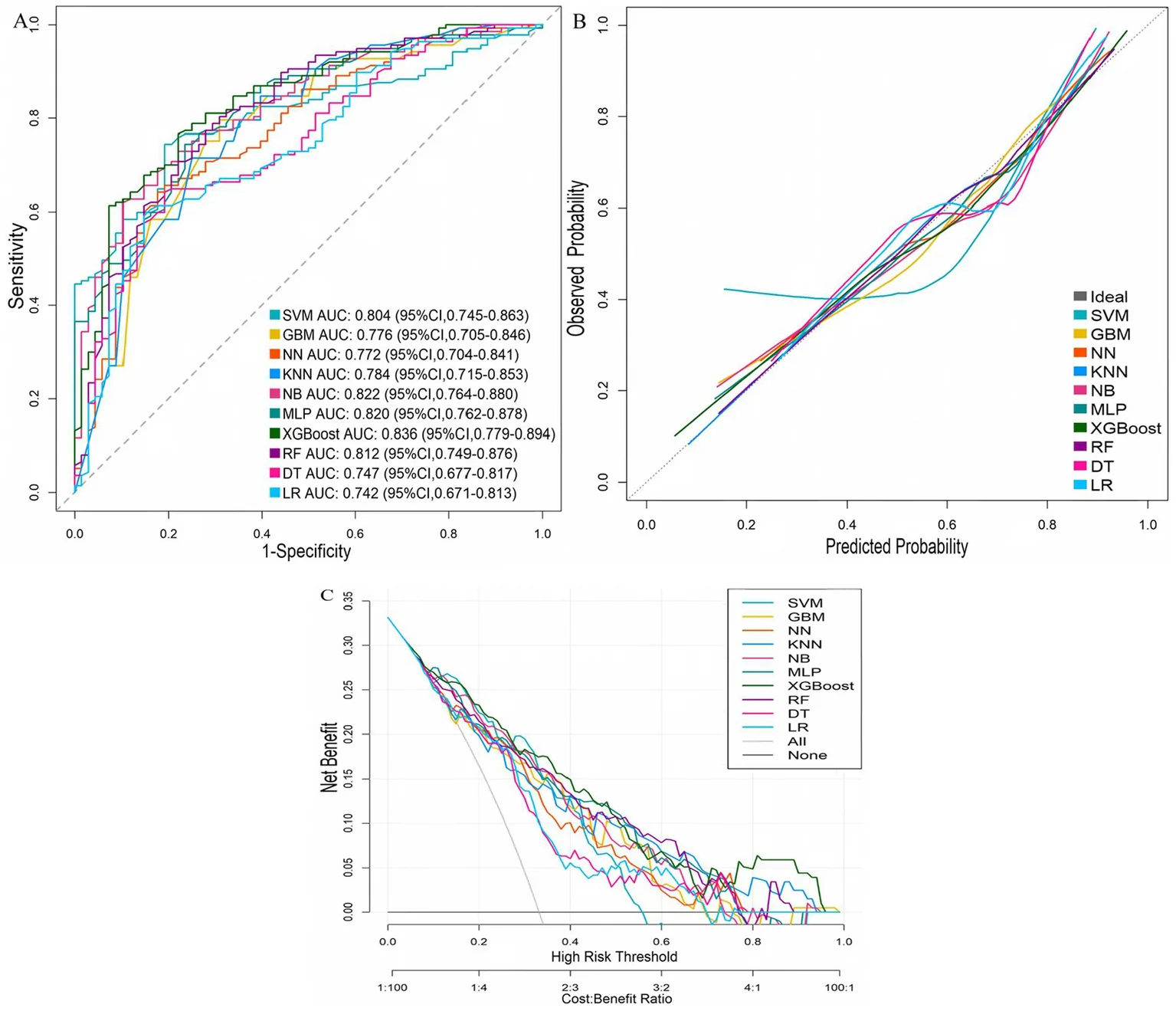
Performance comparison of ten machine learning models. **(A)** Receiver operating characteristic (ROC) curves; **(B)** calibration curves; **(C)** decision curve analysis (DCA).

### SHAP-based interpretability analysis of the optimal XGBoost model

SHAP was employed to visually interpret the optimal XGBoost prediction model, quantifying the contribution and direction of each of the four core predictors to the risk of 6-month postoperative malnutrition (Figure 5). The SHAP feature importance ranking demonstrated that preoperative BMI was the most influential predictor, followed by adjuvant chemotherapy, age, and tumor location. Regarding the directional effects, receipt of adjuvant chemotherapy, older age, lower preoperative BMI, and rectal tumor location were associated with increased predicted malnutrition risk.

**Figure 5:**
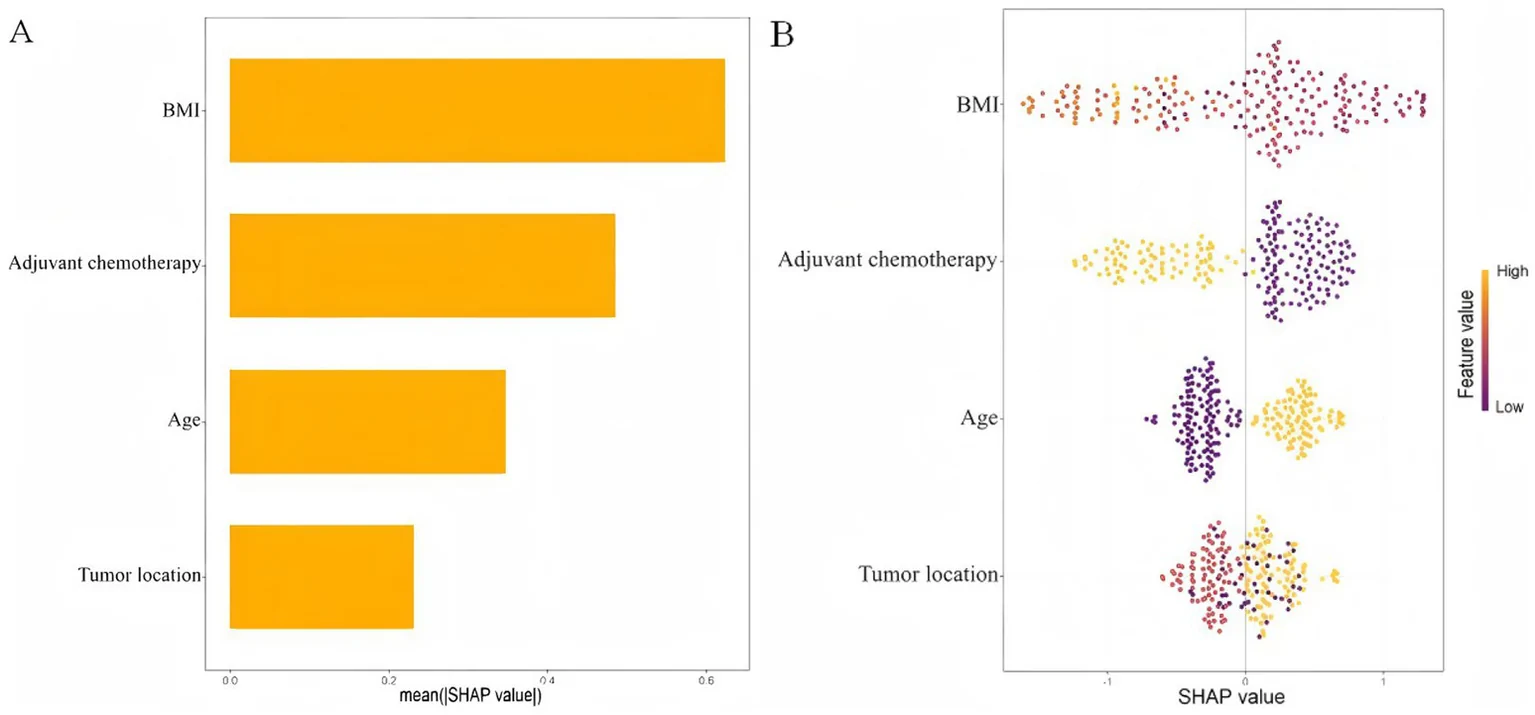
SHAP interpretability of the XGBoost malnutrition prediction model at 6 months post-surgery. **(A)** Feature importance ranking; **(B)** SHAP summary beeswarm plot showing the distribution and directional effects of each predictor. SHAP, SHapley Additive exPlanations; XGBoost, extreme gradient boosting.

## Discussion

Our prospective longitudinal data demonstrate that the overall postoperative weight trajectory after curative resection for colorectal cancer exhibited a time-dependent pattern of gradual decline and subsequent stabilization, decreasing from a peak of 7.8% at hospital discharge (T1) to 2.5% at six months post-surgery (T4). The maximal weight deficit observed at T1 is biologically consistent with the compounded effects of surgery-induced catabolic stress, perioperative fluid shifts, transiently reduced oral intake, and early postoperative gastrointestinal dysfunction. As recovery proceeds, the resumption of oral feeding, resolution of ileus and inflammation, and gradual restoration of digestive function collectively drive a partial rebound in body weight. Notably, however, a residual mean weight loss of 2.5% persisted at six months, indicating incomplete recovery relative to preoperative baseline at the population level. The linear mixed model further revealed that each adjacent time interval (T1 → T2 → T3 → T4) captured statistically significant incremental changes (all *p* < 0.001), implying that this nutritional rehabilitation process is not a random fluctuation but rather a systematically evolving continuum throughout the first six postoperative months.

By applying latent class growth modeling, we successfully disentangled this aggregate trajectory into three distinct and clinically interpretable subgroups with high classification accuracy. The trajectory-specific patterns provide deeper insight into the biological heterogeneity of postoperative recovery. The persistent weight loss group exhibited a sustained approximately 8% deficit without any downward trend, indicating refractory catabolism likely driven by chronic inflammation, persistent malabsorption, or subclinical tumor-related cachexia. This flat trajectory suggests that standard perioperative support is insufficient for this subset, and these patients require prolonged, multimodal nutritional interventions extending well beyond the inpatient period. The gradual recovery group displayed a biphasic pattern—early substantial loss followed by progressive albeit delayed restitution—suggesting that while recovery capacity remains intact, the process is slowed by age-related metabolic decline, comorbid conditions, or transient chemotherapy-related side effects. The rapid recovery group achieved near-baseline weight by three months, reflecting robust preoperative reserves, efficient resolution of surgical inflammation, and preserved gastrointestinal motility, thus requiring only routine follow-up rather than intensified intervention.

The selection of the six-month postoperative time point (T4) as the assessment window for GLIM-defined malnutrition and as the anchor for our machine learning prediction model was based on two primary considerations. First, T4 aligns precisely with the GLIM criteria, which define involuntary weight loss >5% within six months as a core phenotypic criterion, thereby ensuring standardized diagnosis and international comparability. Additionally, the routine contrast-enhanced abdominal CT performed at six months for oncological surveillance permits objective quantification of L3 skeletal muscle mass, a key phenotypic component essential for a comprehensive malnutrition diagnosis. Second, and more fundamentally, our LCGM trajectory data provided robust empirical support for selecting T4. At T3 (three months post-surgery), the gradual recovery group was still in an active phase of weight regain, with a mean weight loss of 2.9%—indicating that the recovery process remained dynamically ongoing. By T4, however, all three trajectory groups had entered a stable plateau, with weight loss percentages showing minimal changes from T3 to T4 (e.g., the mean weight loss of the gradual recovery group decreased from 2.9 to 0.6%). This plateau implies that T4 captures the chronic, stable nutritional status, minimizing confounding from acute perioperative factors such as surgical stress, fluid shifts, delayed gastrointestinal recovery, and chemotherapy-related toxicity. In contrast, an earlier assessment at T3 would still be influenced by these dynamic processes, potentially overestimating malnutrition risk or misclassifying patients who are on a trajectory of recovery. Therefore, T4 provides a more authentic reflection of the chronic disease-related catabolic state, consistent with the etiologic framework of malnutrition within the GLIM criteria.

Previous studies on malnutrition in colorectal cancer have predominantly focused on the preoperative period, with reported prevalence rates ranging from 20 to 50% depending on the diagnostic criteria and study populations employed (8, 17, 21, 22). In contrast, systematic data on long-term postoperative nutritional status remain notably scarce. In our cohort, the overall incidence of GLIM-defined malnutrition at six months post-surgery was 33.2%, a figure that underscores the substantial and persistent nutritional burden faced by colorectal cancer survivors during the extended convalescent phase. This sustained high prevalence is likely driven by multiple interconnected mechanisms: surgically induced alterations in gastrointestinal anatomy and function frequently lead to chronic diarrhea, early satiety, and malabsorption; the cytotoxic effects of adjuvant chemotherapy further compromise nutritional status; and the psychological burden of cancer diagnosis and treatment—including anxiety, depression, and altered eating behaviors—may gradually compromise nutritional status over an extended period (23–26).

Postoperative malnutrition is not merely a biochemical or anthropometric aberration; it is a strong independent predictor of impaired immune surveillance, diminished physical performance, reduced tolerance to and completion rates of adjuvant therapy, and ultimately, inferior long-term survival (27–35). Despite its well-established clinical significance, current practice remains overwhelmingly centered on short-term perioperative nutritional support, while dynamic monitoring and risk-stratification tools for the post-discharge period are conspicuously lacking. This gap is particularly concerning because nutritional deterioration after discharge is often insidious, progressing silently until overt clinical manifestations—such as significant weight loss, functional decline, or treatment intolerance—finally prompt recognition, at which point therapeutic intervention is substantially less effective. Therefore, there exists an urgent and unmet clinical need for early risk identification and proactive nutritional intervention—precisely the rationale that motivated the development of our machine learning-based prediction model.

In contrast to conventional logistic regression, which relies on prespecified linear assumptions and predefined interaction terms, machine learning algorithms offer greater flexibility in capturing complex nonlinear relationships and high-order interactions among predictors without imposing a rigid parametric structure (36). In this study, we developed and compared ten machine learning algorithms based on four core predictors—age, BMI, tumor location, and adjuvant chemotherapy—to predict malnutrition at six months post-surgery. Among the evaluated models, XGBoost demonstrated favorable discriminative ability, excellent calibration, and DCA confirmed that it provided net clinical benefit across a wide range of threshold probabilities. The model can be seamlessly integrated into existing electronic health record systems, potentially enabling automated risk stratification at the individual patient level. Early identification of high-risk patients would allow proactive nutritional interventions—such as intensive dietary counseling, oral nutritional supplements, or early referral to nutrition support teams—to be initiated before overt malnutrition develops, thereby mitigating its downstream consequences on treatment tolerance, functional recovery, and survival (37, 38).

In this study, age emerged as a strong predictor of adverse nutritional outcomes (39). Consistent with our LCGM findings, patients aged > 65 years were overrepresented in the persistent weight loss trajectory and were significantly less likely to achieve rapid recovery. The biological basis for this vulnerability is multifaceted: age-related declines in metabolic reserve, sarcopenia, blunted anabolic responses to nutritional stimuli, reduced digestive and absorptive capacity, and a greater burden of comorbidities collectively compound the surgical stress response and impair the compensatory mechanisms required for postoperative nutritional restitution (40–42).

Tumor location—specifically, rectal cancer versus colon cancer—was another important predictor. Our LCGM results indicated that rectal cancer was independently associated with persistent postoperative weight loss. The pathophysiological basis of this risk is primarily anatomical and functional. Patients undergoing rectal cancer resection are prone to developing LARS, characterized by fecal incontinence, increased stool frequency, urgency, and clustering. These debilitating bowel dysfunctions severely compromise nutrient absorption and disrupt normal dietary intake, creating a sustained barrier to weight recovery (43–45).

Adjuvant chemotherapy was confirmed as a significant predictor of malnutrition and was negatively associated with the rapid recovery trajectory in our LCGM model. Although chemotherapy is indispensable for improving oncological outcomes in patients with stage III and high-risk stage II colorectal cancer, its systemic toxicity poses a direct threat to nutritional status (46). The mechanisms involve chemotherapy-induced gastrointestinal toxicities—including nausea, vomiting, diarrhea, and mucositis—as well as systemic effects such as anorexia and dysgeusia (altered taste perception), all of which collectively impair oral intake and nutrient assimilation (35, 47, 48).

Preoperative BMI emerged as the most influential predictor in the SHAP analysis, yet it did not significantly distinguish between weight trajectory groups in the multinomial logistic regression analysis. This seemingly paradoxical finding can be reconciled by recognizing that BMI reflects baseline nutritional reserve rather than the rate of recovery. Patients with low preoperative BMI start from a depleted baseline; even if their rate of weight recovery is comparable to that of normal-BMI patients, they may still fail to reach normal nutritional thresholds at six months due to their lower starting point. Thus, BMI captures pre-existing vulnerability rather than recovery dynamics. Clinically, patients with low preoperative BMI should be identified as high-risk regardless of their anticipated trajectory, and nutritional prehabilitation initiated before surgery may be particularly beneficial in this subgroup (12, 14).

Several limitations of this study should be acknowledged. First, this was a single-center study with a relatively modest sample size, which may limit statistical power and generalizability. Second, although nested cross-validation was used for internal validation, external validation in independent multi-center cohorts is lacking, and thus the generalizability of the XGBoost model remains to be established. Its favorable predictive performance should therefore be interpreted as preliminary evidence. Third, covariates were selected for the multivariable model based on univariate significance (*p* < 0.05), which may have excluded relevant confounders and made the model somewhat data-driven. Future studies with larger sample sizes should consider including all clinically relevant variables regardless of univariate significance. Fourth, we only assessed nutritional status at six months post-surgery and did not analyze longer-term nutritional changes beyond this time point. Fifth, a substantial proportion of patients were excluded due to incomplete data, which may introduce selection bias, as patients with worse nutritional trajectories might have been disproportionately lost to follow-up. Finally, the assumption that the GLIM etiologic criterion was automatically satisfied for all patients may have inflated the estimated malnutrition prevalence (33.2%), and future studies incorporating objective inflammatory markers are warranted to validate this finding.

In conclusion, this prospective study identified three distinct postoperative weight loss trajectories among colorectal cancer patients and demonstrated that the six-month time point serves as a stable assessment window for GLIM-defined malnutrition. The XGBoost model, incorporating four readily available predictors (age, BMI, tumor location, and adjuvant chemotherapy), showed preliminary favorable predictive performance and clinical utility, enabling early identification of high-risk patients. While further external validation is warranted, this model offers a practical and interpretable tool to support personalized nutritional management in colorectal cancer survivors.

## Data Availability

The original contributions presented in the study are included in the article/Supplementary material, further inquiries can be directed to the corresponding author.
