# Supplementary material for: Dynamic weight loss trajectories and machine learning-based prediction of malnutrition in colorectal cancer patients after curative surgery
Source: Front Nutr. 2026 Oct 21;13:1982995. doi: 10.3389/fnut.2026.1982995 (PMC13730964; doi:10.3389/fnut.2026.1982995)
Supplement: Supplementary file 1 [file Data_sheet_1.pdf]

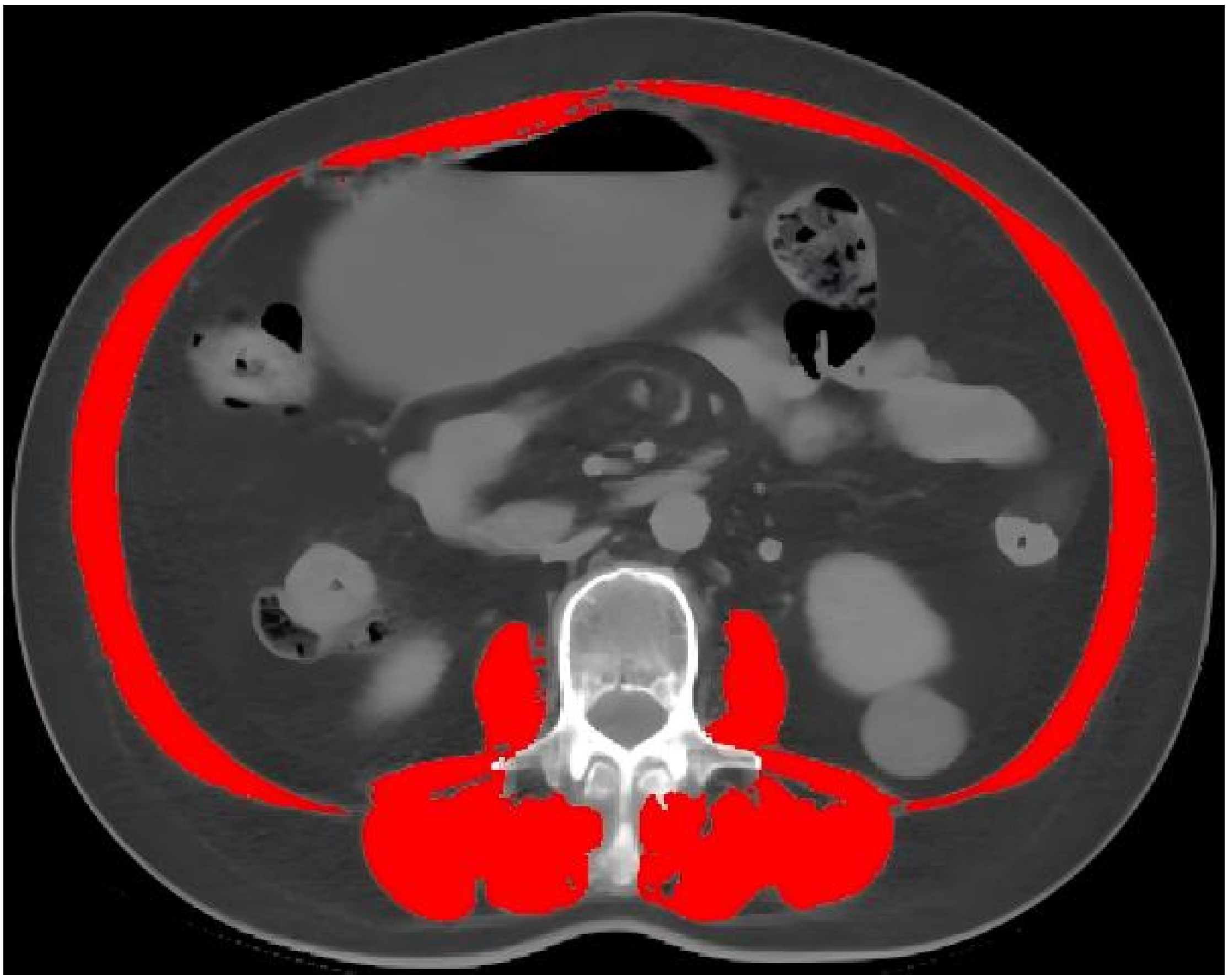

SUPPLEMENTARY FIGURE 1
Computed tomography (CT)-based measurement of skeletal muscle index at the third lumbar vertebra (L3).

SUPPLEMENTARY TABLE 1 Missing data for individual variables (N = 205).

Variables	Missing data
Sex	0 (0.0%)
Age	0 (0.0%)
Hypertension	0 (0.0%)
Diabetes mellitus	1 (0.5%)
Respiratory disease	3 (1.5%)
Smoking	0 (0.0%)
Alcohol consumption	2 (1.0%)
Previous abdominal surgery	1 (0.5%)
Preoperative BMI	1 (0.5%)
Neoadjuvant therapy	0 (0.0%)
Preoperative CEA	8 (3.9%)
Preoperative CA19-9	7 (3.4%)
ASA grade	0 (0.0%)
Surgical approach	0 (0.0%)
Operation time	0 (0.0%)
Intraoperative blood loss	0 (0.0%)

Tumor location	0 (0.0%)
Tumor size	0 (0.0%)
Pathological stage	0 (0.0%)
Differentiation	0 (0.0%)
Lymphovascular invasion	0 (0.0%)
Stoma	0 (0.0%)
Postoperative complications	0 (0.0%)
Adjuvant chemotherapy	0 (0.0%)
Weight at T1	3 (1.5%)
Weight at T2	5 (2.4%)
Weight at T3	7 (3.4%)
Weight at T4	0 (0.0%)

Note: T4 weight data were complete because a 6-month follow-up visit and CT scan were mandatory for study inclusion.

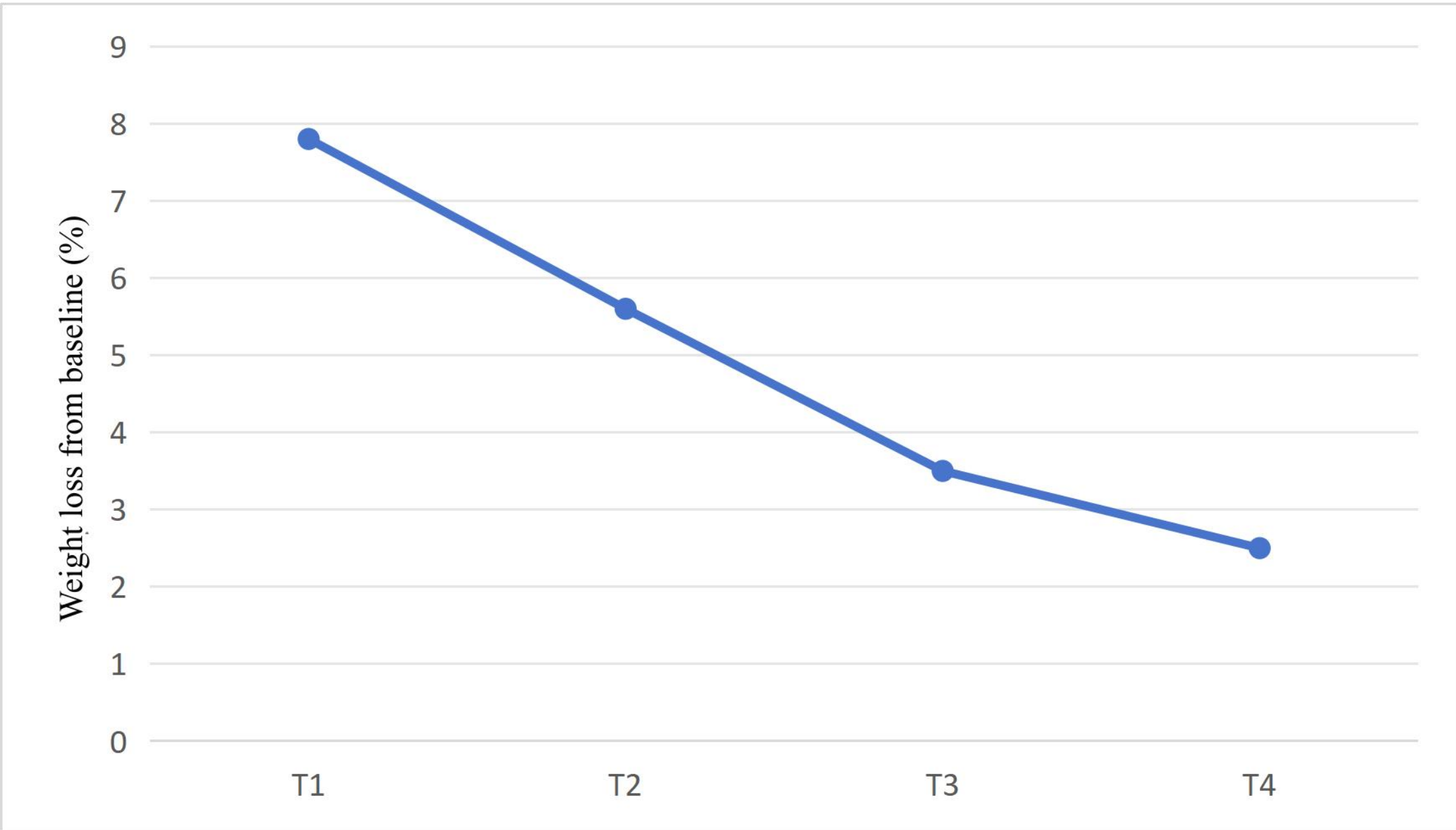

SUPPLEMENTARY FIGURE 2

Overall trend of weight loss percentage over time after colorectal cancer surgery.

SUPPLEMENTARY TABLE 2 Weight loss percentage over time by latent class trajectory.

Group	T1	T2	T3	T4
Persistent weight loss	8.0	7.9	8.1	7.8
Gradual recovery	7.8	6.0	2.9	0.6
Rapid recovery	7.6	3.0	0.4	0.1

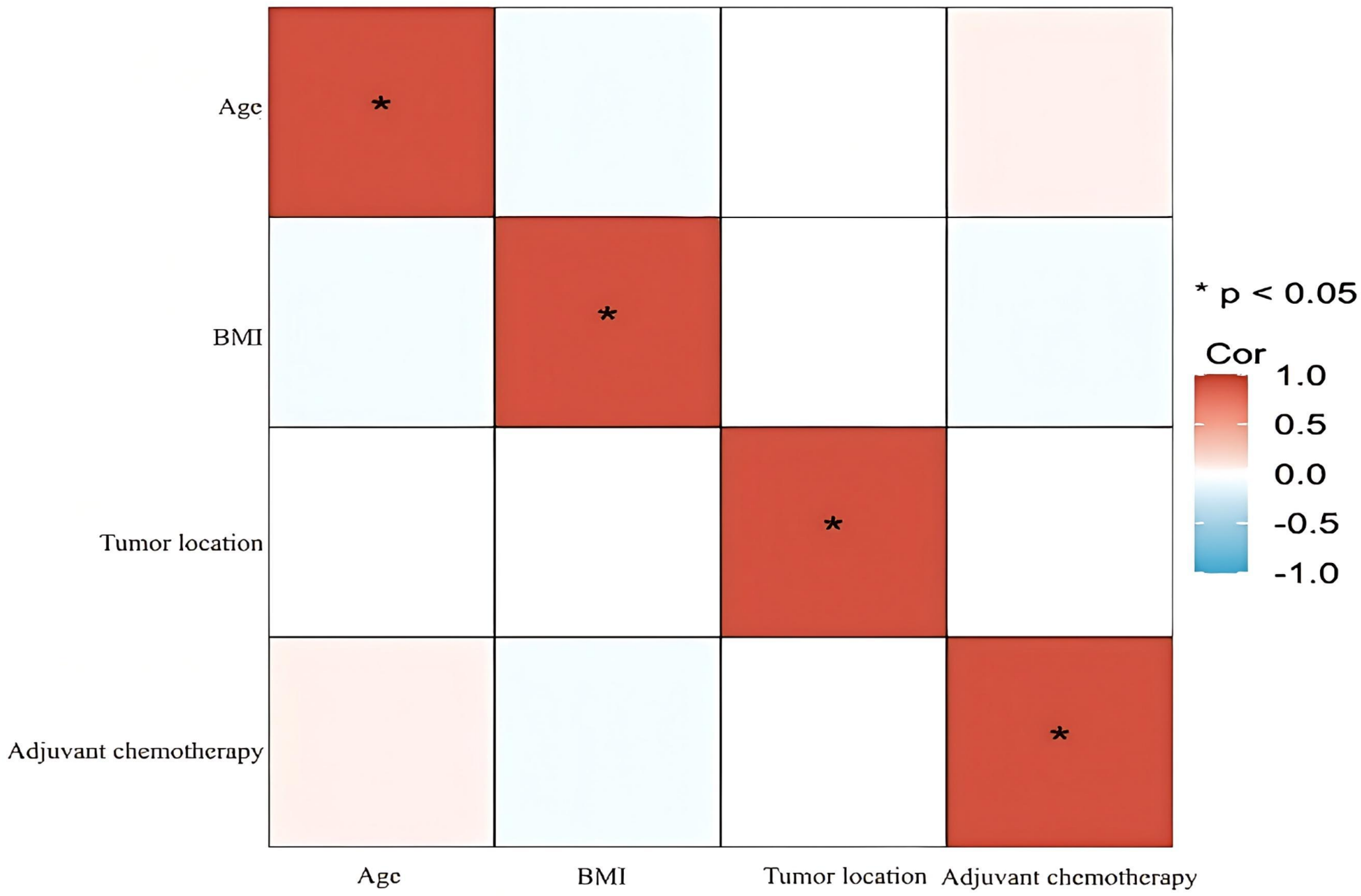

SUPPLEMENTARY FIGURE 3

Correlation heatmap of the four final predictors. BMI, body mass index

SUPPLEMENTARY TABLE 3 Collinearity diagnostics for the predictive variables.

Variable	Tolerance	VIF
Age	0.989	1.012
BMI	0.985	1.015
Tumor location	0.997	1.003
Adjuvant chemotherapy	0.987	1.013

BMI, body mass index; VIF, variance inflation factor.
